# Artesunate Exerts Organ- and Tissue-Protective Effects by Regulating Oxidative Stress, Inflammation, Autophagy, Apoptosis, and Fibrosis: A Review of Evidence and Mechanisms

**DOI:** 10.3390/antiox13060686

**Published:** 2024-06-03

**Authors:** Mingtao Zhu, Yu Wang, Jianwei Han, Yanping Sun, Shuang Wang, Bingyou Yang, Qiuhong Wang, Haixue Kuang

**Affiliations:** 1 Key Laboratory of Basic and Application Research of Beiyao (Heilongjiang University of Chinese Medicine), Ministry of Education, Harbin 150040, China; zmingt180@163.com (M.Z.); w13504560086@163.com (Y.W.); hanjianwei202109@163.com (J.H.); sunyanping@hljucm.edu.cn (Y.S.); wang2066603643@163.com (S.W.); yangbingyou@hljucm.net (B.Y.); 2 School of Traditional Chinese Medicine, Guangdong Pharmaceutical University, Guangzhou 510024, China

**Keywords:** artesunate, antioxidant properties, inflammation, fibrosis, organ protection, molecular mechanisms

## Abstract

The human body comprises numerous organs and tissues operating in synchrony, it facilitates metabolism, circulation, and overall organismal function. Consequently, the well-being of our organs and tissues significantly influences our overall health. In recent years, research on the protective effects of artesunate (AS) on various organ functions, including the heart, liver, brain, lungs, kidneys, gastrointestinal tract, bones, and others has witnessed significant advancements. Findings from in vivo and in vitro studies suggest that AS may emerge as a newfound guardian against organ damage. Its protective mechanisms primarily entail the inhibition of inflammatory factors and affect anti-fibrotic, anti-aging, immune-enhancing, modulation of stem cells, apoptosis, metabolic homeostasis, and autophagy properties. Moreover, AS is attracting a high level of interest because of its obvious antioxidant activities, including the activation of Nrf2 and HO-1 signaling pathways, inhibiting the release of reactive oxygen species, and interfering with the expression of genes and proteins associated with oxidative stress. This review comprehensively outlines the recent strides made by AS in alleviating organismal injuries stemming from various causes and protecting organs, aiming to serve as a reference for further in-depth research and utilization of AS.

## 1. Introduction

In recent years, lower respiratory tract infections, depression, ischemic heart disease, stroke, and cancer have emerged as significant disease burdens across various age groups [1]. Among these, certain diseases precipitate structural and functional impairments or organ and tissue failures. Thus, addressing dysfunctions in various organs and tissues promptly may hold the key to treating a wide array of ailments. Upon invasion by novel coronaviruses, patients have exhibited pathological alterations such as diffuse alveolar injury, myocardial edema, acute tubular injury, and fibrosis in multiple organs [2]. Notably, individuals with cardiovascular diseases like hypertension have shown heightened susceptibility to renal end-organ damage [3]. Furthermore, external factors such as medications and trauma, including commonly used antibiotics like amoxicillin–clavulanic acid, non-steroidal anti-inflammatory drugs, and statins, can inflict harm on organs and tissues [4,5]. Research indicates that organ and tissue damage involves a complex interplay of mechanisms encompassing inflammation, oxidative stress, cellular senescence, apoptosis, autophagy, fibrosis, and metabolic disorders [6]. In addressing the multifaceted nature of diseases marked by diverse pathological changes, natural products with multi-targeted actions have shown promise. Compounds like luteolin [7], silymarin [8], and carvacrol [9] have demonstrated remarkable protective effects on damaged tissues and organs.

As artemisinin has successfully tackled malaria—one of the most infectious, destructive, and debilitating diseases—the exploration of new mechanisms and indications for artemisinin and its derivatives has garnered significant attention to meet clinical demands. Artemisinin semi-synthetic derivatives, including artesunate (AS), artemether, dihydroartemisinin, artether, and arteether, have substantially enhanced the physicochemical properties and drug-forming characteristics of artemisinin while exhibiting ~10 times greater biological potency than artemisinin itself [10]. Among these derivatives, AS has received the most scrutiny due to its more favorable pharmacokinetic–pharmacodynamic profile [11,12]. Compared to compounds like luteolin, silymarin, and carvacrol, AS boasts superior aqueous solubility and oral bioavailability, largely attributable to the hemisuccinate moiety in its structure [13], thereby conferring higher clinical value. Previous reviews addressed either the anti-malarial effects of AS [14] or its therapeutic impacts on respiratory diseases [12], cancer [15], central nervous system disorders [16], viruses [17] skin ailments, and diabetes [18]. To our knowledge, a comprehensive, up-to-date review exclusively focusing on the available reports regarding the organ- and tissue-protective effects of AS has not yet been conducted.

Considering AS’s promising prospects in organ and tissue protection and the scarcity of relevant reviews, this paper aims to compile the most recent advancements of AS in mitigating organ damage caused by various factors and safeguarding organs. With the keywords “Artesunate”, “biological activity of AS”, “organ damage”, “fibrosis”, “anti-oxidation”, “inflammatory injury”, “effects of AS on organ protection”, and “clinical effectiveness”, we conducted an extensive literature search of several databases, including PubMed, Web of Science, and Google Scholar database; the collected articles were categorized by topic and incorporated into this review. A comprehensive understanding of AS’s organ and tissue-protective effects and underlying mechanisms will enhance its capacity to serve as an effective organ and tissue protector and facilitate its clinical application. Furthermore, it may introduce novel perspectives on organ and tissue protection.

## 2. Pharmacological Properties of AS

**Figure 1:**
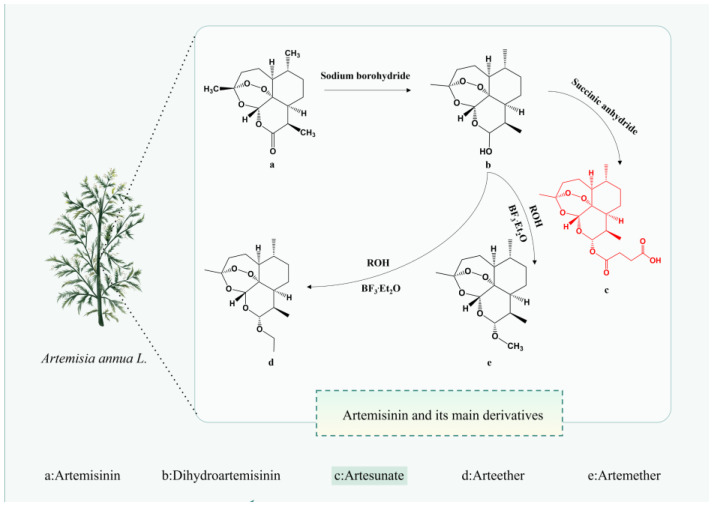
The chemical structure of Artemisinin and its main derivatives. The figure was created with Figdraw.

AS can be administered via various routes including intramuscular, oral, rectal, and intravenous, as well as intranasal, intragastric, and parenteral routes [19]. Among these, the intrarectal route exhibits lower bioavailability and greater inter-individual variability compared to the intravenous route [20]. A systematic review of 50 studies involving AS administration via different routes (oral, rectal, intravenous, and intramuscular) in 1470 subjects, volunteers, and patients revealed that the average concentration (Cav) values achieved via the intravenous route (ranging from 340 to 566 μg/L) significantly surpassed the half-maximal effective concentration (EC50) in vivo (EC50 value of 9.92 μg/L). Moreover, Cav values for the oral route ranged from 22 to 123 μg/L, underscoring the superior relevance of the intravenous route, even in the presence of resistance leading to elevated EC50 values [21]. Hence, the therapeutic efficacy of AS administration routes ranks as intramuscular > rectal > oral. In another study [22], intranasal administration of AS in a female CBA/J mouse model with cerebral malaria improved survival rates, led to the degradation of AS to dihydroartemisinin (DHA) within 15-h post-administration, and reduced blood parasitemia after 24 h. This suggests that the intranasal route represents the most expedient means to deliver the drug to the brain promptly [23]. Regardless of the administration route, AS undergoes rapid metabolism to DHA (Figure 1) in the body and is excreted within a short timeframe.

**Figure 2:**
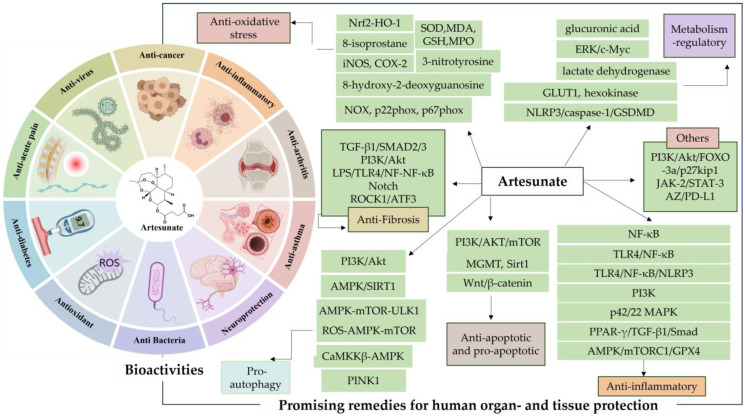
Artesunate (AS) bioactivities. The figure was created with MedPeer.cn (accessed on 1 April 2024).

AS is well known as the first-line therapeutic agent for severe malaria, and has garnered extensive attention from researchers due to the discovery of its diverse pharmacological activities in recent years (Figure 2). These activities include regulating blood glucose and lipids [24], providing neuroprotection and analgesia [25], inducing pro-apoptosis, inhibiting angiogenesis, and suppressing osteoclastogenesis [26], as well as displaying anti-inflammatory [27], antiviral [28], and antibacterial effects [29]. Such findings underscore its therapeutic potential across various diseases such as obesity [30], diabetes mellitus [31,32,33], Alzheimer’s disease [34], chronic pain [35], asthma [36], and cancer [37,38].

Moreover, AS exhibits excellent physicochemical and pharmaceutical properties, with fewer adverse effects and reduced susceptibility to drug resistance, thereby demonstrating remarkable clinical benefits. Encouragingly, studies investigating the protective effects of AS on multiple tissues and organs have shown compelling results, demonstrating significant ameliorative and salvage capabilities in various models of organ damage and disease. AS achieves this by modulating multiple mechanisms of injury and targeting molecular pathways, positioning it as one of the most promising drug candidates in biomedicine and pharmacology.

## 3. Protective Effects and Action Mechanisms of AS

### 3.1. AS Protects Organs and Tissue through Anti-Inflammatory Effect

Inflammation plays a pivotal role in the pathogenesis of numerous diseases, making it a significant therapeutic target. Among the key mediators of inflammation, the nuclear factor-κB (NF-κB) family of transcription factors stands out as a central regulator [39]. Studies investigating the treatment of distal middle cerebral artery occlusion with AS have demonstrated its ability to reduce levels of pro-inflammatory cytokines, such as tumor necrosis factor-α (TNF-α) and interleukin-1β (IL-1β), thus attenuating ischemic injury and inflammation post-cerebral ischemia by inhibiting the activation of the NF-κB signaling pathway [40]. Additionally, AS exhibited significant gastroprotective effects in rats, alleviating gastric mucosal injury through its anti-inflammatory and antioxidant properties, and modulating the NF-κB pathway [41].

Activation of toll-like receptors (TLRs) has been shown to promote the nuclear translocation of NF-κB and the upregulation of inflammatory enzymes and cytokines [42]. Notably, studies have demonstrated that AS, at a dose of 80 mg/kg, effectively inhibits the TLR4/NF-κB pathway, reducing cerebral infarct volume, improving neurological deficits, and mitigating cerebral neuroinflammation [43]. Moreover, in the context of traumatic brain injury, AS treatment at a dose of 30 mg/kg has been shown to inhibit the protein levels of NF-κB, NLRP, GFAP, and Iba-1, while modulating the levels of neurotrophic factors such as BDNF, GDNF, and NT-3 [44]. Furthermore, Zhao et al. showed that AS downregulated hepatic enzymes, interferon (IFN)-γ, TNF-α, interleukins (IL)-1β, IL-6, and IL-17, as well as key signaling molecules including extracellular signal-regulated kinase (ERK), c-Jun N-terminal kinase (JNK), p38 mitogen-activated protein kinase (p38), nuclear factor-κBα (IκBα), and phosphorylated NF-κB p65 in concanavalin A-induced autoimmune hepatitis mice. This modulation of inflammatory response pathways attenuates hepatic injury through the NF-κB and mitogen-activated protein kinase (MAPK) signaling pathways [45].

**Figure 3:**
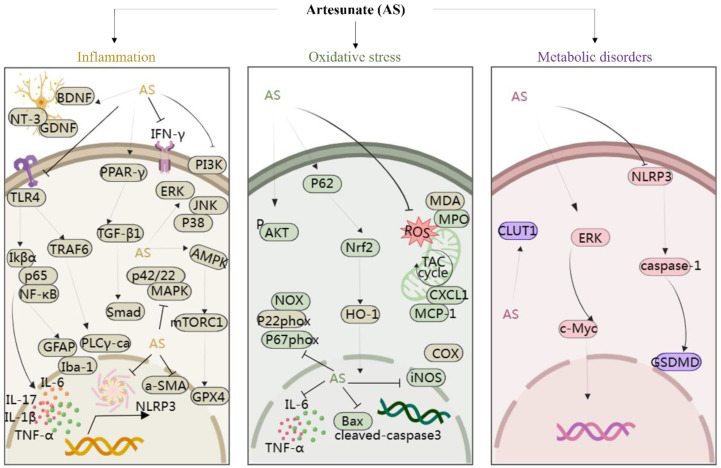
The protection of organ- and tissue mechanism of AS against inflammatory storm, oxidative stress, and metabolic disorders. The figure was created with MedPeer.cn. (accessed on 1 April 2024). (HO-1, Heme oxygenase-1; IL, interleukin; TNF, tumor necrosis factor; Nrf2, Nuclear factor-E2-related factor 2; NF-κB, Nuclear factor kappa B; PI3K, Phosphoinositide 3-kinase; PPARγ, Peroxisome proliferator-activated receptor γ; ERK1/2, extracellular signal-regulated kinase 1/2; Bax, Bcl-2-associated X protein; BDNF, Brain-derived neurotrophic factor; JNK, c-Jun N-terminal kinase; MAPK, Mitogen-activated protein kinase; mTOR, Mammalian target of rapamycin; TLR, Toll-like receptor; p38MAPK, p38 mitogen-activated protein kinase; AMPK, adenosine monophosphate-activated protein kinase; JAK, Janus kinase; GLUT, glucose transporter; COX-2, cyclooxygenase-2; NLRP3, nucleotide-binding domains and leucine-rich repeat pyrin domains containing 3; caspase, Cysteine aspartic acid specific protease; TRAF6, TNF receptor–associated factor 6; NT3, neurotrophins-3; GDNF, glial cell derived neurotrophie factor; iNOS, inositol; GPX4, Glutathione Peroxidase 4; MDA, malondialdehyde; MPO, myeloperoxidase; α-SMA, α-smooth muscle actin; GSDMD, Gasdermin-D; IKB-α, Human nuclear factor kappa B suppressor protein alpha; PLC, Phospholipase C; GFAP, glial fibrillary acidic protein).

Moreover, studies have suggested that AS exerts protective effects on rat glomerular mesangial cells [46] and modulates arterial inflammatory responses through the TLR4/NF-κB/NLRP3 inflammasome pathway [47], highlighting its potential therapeutic role in diabetic nephropathy and atherosclerosis. In addition, Yin et al. discovered that AS significantly suppressed the phosphorylation of IκBα and NF-κB p65, thus reducing intestinal inflammation in mice [48]. In a study involving cigarette smoke-induced airway remodeling in chronic obstructive pulmonary disease (COPD) rats, AS demonstrated inhibition of airway inflammation, treatment of airway remodeling, and downregulation of α-smooth muscle actin (α-SMA) and cytosolic protein D1 levels through the PPAR-γ/TGF-β1/Smad signaling pathway [49]. In addition, Xie et al. revealed that AS exerted neuroprotective effects by suppressing inflammation via the AMPK/mTORC1/GPX4 pathway [50]. Further, PI3K and p42/22 MAPK are known to have pro-inflammatory effects, and AS reversed the inflammatory cascade in a COPD model by inhibiting the expression of PI3K and p42/22 MAPK [51]. Notably, AS at a dose of 10 mg/kg, administered intraperitoneally, inhibited the levels of TLR4/TRAF6 and downstream PLCγ1-Ca, ameliorating lipopolysaccharide-induced (LPS; 5 mg/kg, administered intraperitoneally) inflammatory bone loss in vivo [52]. In summary, AS mitigates inflammatory responses by modulating the levels of various inflammatory mediators and targeting signaling pathways such as NF-κB, NLRP3, TLR4, PI3K, p42/22 MAPK, PPAR-γ/TGF-β1/Smad, and AMPK/mTORC1/GPX4. This multifaceted approach enables AS to exert protective effects on various organs and tissues including the brain, nerves, stomach, lungs, bones, liver, kidneys, and blood vessels, thereby playing a crucial protective role (Figure 3).

### 3.2. AS Protects Organs and Tissue through Antioxidative Stress Effect

The imbalance between oxidative and antioxidant systems can instigate the onset and progression of various diseases such as heart failure [53] and Alzheimer’s disease [54], while also causing significant damage to organs and tissues. Oxidative stress triggers the release of reactive oxygen species (ROS), activating diverse signaling pathways and influencing gene and protein expression. Notably, the nuclear factor erythroid 2-related factor (Nrf2) emerges as a crucial redox-sensitive transcription factor, and its activation has been recognized as an effective therapeutic strategy against oxidative stress-related diseases [55].

Studies have revealed that AS effectively inhibits the expression of oxidative damage markers such as 8-isoprostane, 8-hydroxy-2-deoxyguanosine, and 3-nitrotyrosine, while downregulating the levels of NOX, p22phox, and p67phox. Additionally, AS modulated the expression of antioxidants such as superoxide dismutase and catalase, ultimately improving asthma through the activation of Nrf2 [56]. Heme oxygenase-1 (HO-1), a key downstream target gene regulated by Nrf2, plays a vital role in inducing the organism’s antioxidant response. Ji et al. underscored AS’s mitigating effect on lung injury, associated with downregulation of MDA, MPO, IL-1β, TNFα, CXCL1, MCP-1, Bax, and cleaved Caspase3 protein levels, alongside upregulation of P-AKT and HO-1 proteins [57]. Furthermore, AS upregulates Nrf2 and HO-1 levels to alleviate acute lung injury in mice with LPS-induced lung injury [58].

Cao et al. reported that activation of Nrf2 and upregulation of HO-1 expression by AS contribute to the downregulation of cyclooxygenase-2 (COX-2) and nitric oxide synthase (iNOS) levels, thereby exerting a protective effect in sepsis-associated lung injury [59]. Moreover, AS activated the p62/Nrf2 pathway to downregulate ROS levels, inhibiting osteoclastogenesis and ameliorating bone erosion in rheumatoid joints [60]. Notably, in a separate study, AS treatment at doses of 5 and 15 mg/kg reduced depression and anxiety-like behaviors in rats, primarily attributed to AS’s antioxidant effect [61]. Taken together, AS emerges as a potential candidate for asthma, rheumatoid arthritis, lung injury, depression, and anxiety, exerting a protective effect by targeting signaling pathways associated with oxidative stress and regulating the expression of related upstream and downstream proteins (Figure 3).

### 3.3. AS Protects Organs and Tissue by Regulating Metabolism

Various diseases, such as obesity, tumors, and airway inflammation, disrupt the body’s metabolism, often involving aberrant sugar consumption. Studies have indicated that AS plays a pivotal role in regulating metabolic diseases. For instance, Zhang et al. discovered that AS could impede the growth of non-small cell lung cancer (NSCLC) cells by modulating glucose metabolism through the ERK/c-Myc pathway. They demonstrated that AS and its metabolite DHA reduced glucose uptake, ATP production, and lactate secretion, while also downregulating the levels of glycolytic metabolism-related enzymes and transporter proteins, including glucose transporter protein GLUT1, hexokinase, and lactate dehydrogenase in a mouse model of Lewis lung carcinoma. These results showed the protective effect of AS against tumor growth by regulating glucose metabolism in a time- and dose-dependent manner [62].

Furthermore, AS has shown promise in ameliorating metabolic alterations in airway inflammation. Ho et al. observed the upregulation of metabolites such as sterols, phosphatidylcholine, galactose, glucose, and glucuronic acid, coupled with the downregulation of pro-inflammatory metabolites in the arginine–proline metabolic pathway in asthmatic mice following AS treatment [63]. In atherosclerotic mice, AS corrected dysfunction in crucial metabolic pathways [64]. Additionally, in streptozotocin-induced diabetic mice, AS improved blood glucose levels and protected pancreatic β-cells by inhibiting the NLRP3/caspase-1/GSDMD pathway, suggesting its potential as a promising artemisinin derivative for diabetes treatment [65]. In summary, AS exhibits a notable capacity to regulate metabolism (Figure 3), offering therapeutic potential across various metabolic disorders, including cancer, airway inflammation, atherosclerosis, and diabetes.

### 3.4. AS Protects Organs and Tissue through Anti-Fibrotic Effect

**Figure 4:**
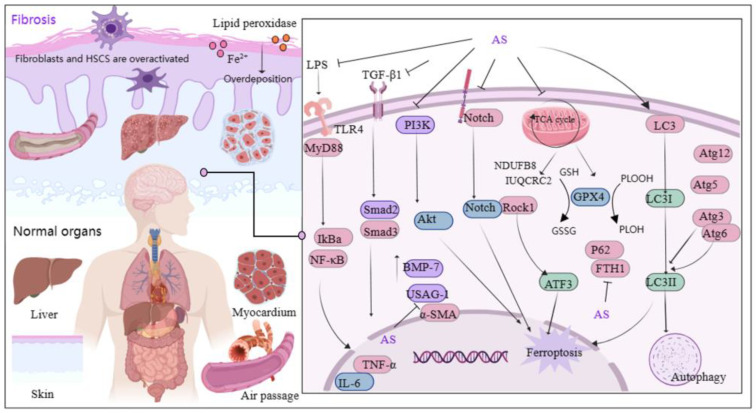
Mechanism of AS on organ and tissue protection from fibrosis. The figure was created with MedPeer.cn (accessed on 3 April 2024). (IL, interleukin; TNF, tumor necrosis factor; PI3K, Phosphoinositide 3-kinase; TLR, Toll-like receptor; GPX4, Glutathione Peroxidase 4; α-SMA, α-smooth muscle actin; MyD88, myeloid differentiation factor 88; LC3, microtubule-associated protein light chain 3; NF-κB, Nuclear factor kappa B; IKB-α, Human nuclear factor kappa B suppressor protein alpha; GSH, Glutathione; Atg3/6/5/12, autophagy related Gene; Akt, Protein kinase B; Rock1, Rho Associated Coiled-Coil Containing Protein Kinase 1; ATF3, activating transcription factor 3 Gene; ferritin heavy chain (FTH1); BMP-7, bone morphogenetic protein-7; USAG-1, uterine sensitization-associated gene-1).

Fibrosis primarily arises from excessive deposition of extracellular matrix and overactivation of fibroblasts, a phenomenon that is globally distributed and increasing in prevalence each year [66]. Transforming growth factor-β1 (TGF-β1) plays a significant role in fibrosis [67], and the TGF-β1/Smads pathway is considered pivotal in anti-fibrotic therapy [68,69]. Recently, AS demonstrated effectiveness in treating fibrotic diseases (Figure 4). Studies showed that AS significantly reduces fibroblast activation and suppresses the expression of pro-fibrotic genes [70]. Furthermore, AS’s mechanism in alleviating renal interstitial fibrosis involved increasing bone morphogenetic protein-7 levels and suppressing uterine sensitization-associated gene-1 expression [71]. AS administration has notably ameliorated bleomycin-induced pulmonary fibrosis in Sprague Dawley rats by reducing the levels of profibrotic molecules and downregulating the expression of TGF-β1, Smad3, heat shock protein 47, α-smooth muscle actin, and collagen type I [72]. The potential molecular mechanism of AS against pulmonary fibrosis is also associated with downregulating α-SMA and type IV collagen, as well as inhibiting the Notch signaling pathway [73].

Inflammation plays a central role in fibrosis development, and persistent inflammatory stimulation can lead to organ or tissue sclerosis. Therefore, targeting inflammatory signaling pathways can aid in fibrosis treatment. Lai et al. discovered that AS downregulates the levels of toxin, TNF-α, and IL-6-associated cytokines by reducing inflammatory infiltration in hepatic fibrotic rats following AS intervention with 28.8 mg/kg. AS also down-regulated the protein and mRNA levels of TLR4, α-SMA, myeloid differentiation factor 88, and TGF-β1. Additionally, AS significantly inhibited the nuclear translocation of NF-κB p65. These findings suggest that AS’s regulation of the LPS/TLR4/NF-κB signaling pathway is a relevant mechanism in attenuating inflammation and rescuing liver fibrosis [74]. Another study demonstrated that AS fights liver fibrosis by inhibiting inflammation and collagen fiber deposition [75].

Mitochondria serve as the orchestrators of cell health and act as the gatekeepers of cell death [76]. They primarily regulate iron metabolism and oxidative metabolism and are crucial for the cellular generation of ROS [77]. However, iron overload and excessive deposition of lipid peroxidation lead to the release of large quantities of ROS, disrupting the balance between their production and removal and triggering cellular iron death [78]. Iron death, a novel form of programmed cell death distinct from apoptosis, necrosis, and autophagy, is extensively involved in fibrosis development, suggesting that intervening in iron death may emerge as a therapeutic strategy for fibrosis [79,80]. Recent reports indicated that AS reduces fibroblast activation by inhibiting the TGF-β1/SMAD2/3 and PI3K/Akt pathways. Moreover, it induced mitochondria-dependent iron death by suppressing glutathione peroxidase 4 (GPX4) expression, thereby ameliorating ocular fibrosis [81]. 

Studies have highlighted the pivotal role of hepatic stellate cells (HSCs) in hepatic fibrosis initiation. Activation of numerous HSCs is a hallmark of liver fibrosis, and inhibiting their activation and proliferation can reverse this pathological process [82]. In a mouse model of carbon tetrachloride-induced hepatic fibrosis, AS intervention decreased HSC viability while increasing iron accumulation and lipid peroxidation levels. Additionally, AS upregulated the levels of autophagy-associated proteins LC3 (i.e., microtubule-associated protein light chain 3), Atg3, Atg5, Atg6/beclin1, and Atg12, while downregulating the levels of p62 and ferritin heavy chain (FTH1). These findings suggest that AS’s anti-hepatic fibrosis effects are primarily mediated through ferritin autophagy regulation and induction of HSC iron death [83]. Wang et al. demonstrated that AS induced HSC iron death via the ROCK1/ATF3 axis [84]. Furthermore, another study revealed that AS attenuated schistosome-induced liver fibrosis by down-regulating NDUFB8 and UQCRC2 in mitochondria, thereby inhibiting HSC activity [85]. Significantly, fibrosis is characterized by tissue and organ scarring and sclerosis, and research has indicated that AS can inhibit choroidal neovascularization and improve fibrotic scarring by modulating mononuclear phagocyte recruitment [86]. In short, AS’s protective effect on organs through its anti-fibrotic properties is partly attributed to its ability to suppress fibroblast activation, inflammation, collagen fiber deposition, and promotion of iron death. Additionally, AS modulates related pathways, including TGF-β1/SMAD2/3, PI3K/Akt, LPS/TLR4/NF-κB, Notch, and ROCK1/ATF3.

### 3.5. AS Protects Organs and Tissue by Affecting Apoptosis 

Cell injury often culminates in cell death, with apoptosis being the predominant mechanism of cell demise in mammalian cell cultures. Apoptosis is intricately regulated by intrinsic genetic mechanisms and apoptosis-related genes. Among these, the cysteine–aspartic protease (caspase) and Bcl-2 (B-cell lymphoma 2) protein families play crucial roles in apoptotic pathway regulation. Notable members include caspase-3, caspase-8, caspase-10, Bcl-2, and Bcl-xL (B-cell extra-large lymphoma), which govern apoptotic signaling [87]. The PI3K pathway triggers downstream activation of Akt, inhibiting pro-apoptotic factors like Bax, caspase-9, and GSK-3. Studies have indicated that AS can confer protective effects on damaged organs or tissues across various disease models by modulating the expression of pro-apoptotic factors and enhancing levels of anti-apoptotic factors. For instance, Zhang et al. illustrated that AS mitigates nerve damage by suppressing apoptosis through the PI3K/AKT/mTOR signaling pathway in rats with diabetic peripheral neuropathy induced by a high-fat diet and streptozotocin [88]. In the context of LPS-induced acute lung injury, AS attenuated apoptosis by upregulating lung levels of p-mTOR, p-Akt, and PI3K, while downregulating the levels of cleaved caspase-3, TNF-α, and IL-6, thus shielding the lung from injury. These effects are closely associated with mTOR/AKT/PI3K pathway activation [89]. Interestingly, in lung adenocarcinoma cell lines ASTC-a-1 and A549, AS induces apoptosis through a Bak-mediated intrinsic pathway, which is caspase-dependent rather than caspase-independent [90].

Two crucial enzymes, Sirt1 and O6-methylguanine-DNA-methyltransferase (MGMT), play pivotal roles in apoptosis regulation. Sirt1, a 747-amino acid-long NAD-dependent deacetylase, exerts its apoptotic regulatory function mainly through the deacetylation of key proteins. In a study by Liu et al., it was demonstrated that the upregulation of SIRT1 levels was critical for AS-mediated inhibition of lung cell apoptosis, alleviation of lung dysfunction, and suppression of neutrophil infiltration [91]. MGMT is a DNA repair enzyme pivotal for preventing mutations in cellular DNA and repairing DNA damage. The Wnt signaling cascade has been implicated in cancer cell development, including processes such as proliferation, migration, and drug resistance, by regulating MGMT levels [92,93]. Ismail et al. reported that AS modulated Wnt/β-catenin signaling and interfered with MGMT function, resulting in enhanced induction of synergistic DNA damage and apoptosis [94]. Another study demonstrated that AS suppressed the levels of necrotic apoptotic signals RIPK1, RIPK3, and MLKL in mouse kidneys by downregulating the levels of inflammatory factors such as IL-1β, IL-6, and TNF-α, along with inflammatory signals like iNOS and NF-κB. This establishes AS as a promising therapeutic option for ameliorating cisplatin-induced acute kidney injury [95].

**Figure 5:**
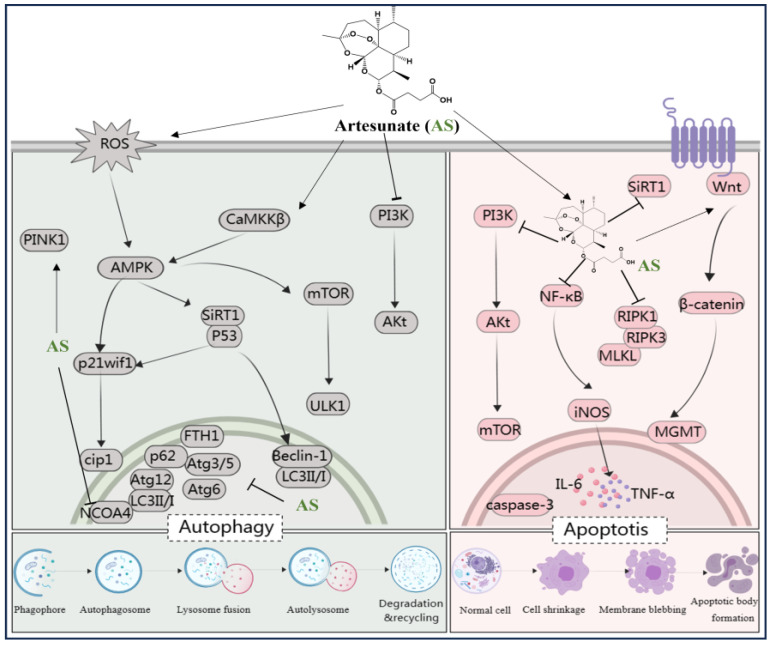
AS regulates various signaling pathways involved in autophagy and apoptosis. The figure was created with MedPeer.cn (accessed on 1 April 2024). (IL, interleukin; TNF, tumor necrosis factor; NF-κB, Nuclear factor kappa B; PI3K, Phosphoinositide 3-kinase; PPARγ, Peroxisome proliferator-activated receptor γ; mTOR, Mammalian target of rapamycin; caspase, Cysteine aspartic acid specific protease; iNOS, inositol; LC3, microtubule-associated protein light chain 3; Atg3/6/5/12, Beclin-1, autophagy related Gene; Akt, Protein kinase B; ROS, reactive oxygen species; ferritin heavy chain (FTH1); AMPK, adenosine monophosphate-activated protein kinase; SIRT-1, Silent information regulator 1; ULK1/2, serine/threonine kinase 1/2; CaMKK, calcium/calmodulin kinase; nuclear receptor co-activator 4 (NCOA4); O6-Methylguanine-DNA-methyltransferase (MGMT); necrotic apoptotic signals RIPK1, RIPK3, and MLKL).

Taken together, AS exhibits both anti-apoptotic and pro-apoptotic effects across different disease models. By targeting key signaling pathways (e.g., PI3K/AKT/mTOR and Wnt/β-catenin) and enzymes (e.g., Sirt1 and MGMT), AS shows promise for therapeutic intervention in apoptosis-related disorders, including diabetic neuropathy, lung injury, cancer, and cisplatin-induced kidney injury (see Figure 5).

### 3.6. AS Protects Organs and Tissue through Pro-Autophagy Effect

Autophagy represents a cytolytic metabolic mechanism crucial for maintaining cellular homeostasis. It can be triggered by various factors such as nutrient deprivation, hypoxia, organelle damage, and proteotoxic aggregates [96,97]. Dysfunctional autophagy, whether deficient or excessively active, is pervasive in numerous human diseases. It encompasses a multifaceted process governed by several important signaling pathways, primarily involving hypoxic, amino acid, serum, and glucose deprivation stress signals [98]. Notably, amino acid depletion triggers the PI3K/serine–threonine-specific protein kinase (AKT)/mTOR pathway [99], while glucose scarcity activates the adenylate-activated protein kinase (AMPK) pathway [100]. Further, genes like LC3, Atg3/5, Atg6/Beclin1, Atg12, p62/53/21, among others, actively regulate autophagy. Targeted autophagy agents have attracted scientific attention and recognition [101,102]. In rats with type 2 diabetes mellitus (T2DM) induced through a high-fat diet and streptozotocin, administration of AS mitigated salivary gland hypoplasia in desiccation syndrome. It achieved this by modulating the PI3K/Akt pathway, thus inhibiting apoptosis and autophagy in T2DM rats [103].

The mammalian target of rapamycin (mTOR) serves as a pivotal regulator of autophagy. AS has been identified as a treatment for knee fibrosis by inhibiting mTOR signaling, thus inducing Beclin-1-mediated autophagy, and mitigating the adverse effects of knee joint surgery [104]. In addition, intervention with AS has been shown to reverse increased retinal thickness in diabetic rats, with the activation of the AMPK/SIRT1 pathway promoting retinal autophagy by upregulating Beclin-1 and LC3II/I levels and downregulating p62 levels [105]. In vitro experiments demonstrated that AS, in combination with metformin, triggers autophagy-dependent cell death in glioblastoma by disrupting the ROS-AMPK-mTOR axis [106]. Kong et al. emphasized the upregulation of LC3 levels and the downregulation of Atg3/5, Atg6/Beclin1, Atg12, p62, FTH1, and nuclear receptor co-activator 4 (NCOA4) mRNA levels, which are involved in the anti-hepatocellular fibrosis effects of AS [83]. Another study reported that AS could restore autophagy by modulating the CaMKKβ-AMPK cascade, thereby rescuing sepsis-induced impairment of reactive function in intrinsic immune cells [107]. Additionally, AS can upregulate the levels of p53 and p21waf1/cip1 to induce autophagy, alleviating surgery-induced epidural fibrosis [108]. Zhang et al. revealed that AS induced mitochondrial autophagy through activation of the PINK1-dependent pathway, enhancing the activity of anti-cervical cancer Hela cells [109]. Furthermore, AS upregulated ROS and activated the AMPK-mTOR-ULK1 pathway, inducing autophagy followed by apoptosis activation in human bladder cancer cells [110]. These findings suggest that influencing autophagy-related pathways such as PI3K/Akt, AMPK/SIRT1, AMPK-mTOR-ULK1, ROS-AMPK-mTOR, CaMKKβ-AMPK, p53/p21waf1/cip1, and PINK1 may represent a potential mechanism for treating diabetic complications, neuroblastoma, fibrosis, sepsis, cervical cancer, and protecting salivary glands, knee joints, liver, retina, and immune cells using AS (Figure 5).

### 3.7. Other Protective Effects of AS

AS demonstrated therapeutic and protective effects by modulating the proliferation, apoptosis, and differentiation of stem cells, as primarily elucidated by the research of Zhang et al. and Luan et al. Adult stem cells are characterized by self-renewal, high proliferation, and differentiation capacities, crucial for tissue and organ homeostasis and repair following damage [111]. Among these, neural stem cells (NSPCs) are versatile neural cells capable of differentiating into neurons and astrocytes, maintaining the structural and functional integrity of the central nervous system [112,113,114,115]. Cerebrovascular diseases result in irreversible neuronal damage, and NSPC proliferation facilitates the release of nerve growth factors, aiding in neuronal repair and neural circuitry reconstruction. Thus, regulating and activating NSPCs are pivotal for ameliorating cerebrovascular diseases [116,117,118]. In this regard, Zhang et al. observed a significant reduction in the area of cerebral infarction and upregulation of Nestin levels, a marker of NSPC proliferation, following AS intervention at 150 mg/kg, suggesting a notable enhancement in NSPC proliferation. Moreover, the PI3K/Akt/FOXO-3a/p27kip1 signaling pathway was identified as critical in enhancing NSPC proliferation in the infarcted cortex [119]. Further, Luan et al. established an ischemic stroke model, demonstrating that AS substantially promoted NSPC proliferation and differentiation, reduced NSPC apoptosis by inhibiting the JAK-2/STAT-3 signaling pathway, and upregulated differentiation-related molecules such as doublecortin and proliferating cell nuclear antigen [120].

Over time, the organism experiences a decline in both the number and functionality of stem cells, ultimately culminating in senescence. Cellular senescence not only precipitates organ failure and diminished tissue function but also exacerbates the onset and progression of chronic diseases. Certain chemotherapeutic agents, such as adriamycin, irinotecan, and fluorouracil, have been noted for their cytotoxic effects, which can induce cellular senescence, limiting their clinical applications [121,122]. In a mouse model of irinotecan-induced cellular senescence, AS demonstrated the ability to delay senescence by inhibiting mTOR signaling. Moreover, AS exhibited the potential to ameliorate irinotecan-induced intestinal inflammation and injury by downregulating the expression of inflammatory factors [123]. Another study highlighted AS intervention’s significant attenuation of fluorouracil-induced intestinal inflammation through the inhibition of p38MAPK and NF-κB signaling pathways. Additionally, AS improved intestinal injury and regulated aging by suppressing mTOR signaling, leading to the downregulation of senescence-related proteins and genes such as p53, p16, and p21 [124].

Meanwhile, recent research indicates that the protective effects of AS extend to immunomodulation. In a study by Cao et al., a model of NSCLC was constructed. Through molecular docking and cellular thermal displacement analysis, AS was found to inhibit NSCLC growth, increase infiltration of CD8+ T cells, and decrease levels of transcriptional co-activator with a PDZ-binding motif (TAZ) and programmed death ligand 1 (PD-L1) in NSCLC. This suggests that AS enhances anti-tumor immunity and mitigates lung cancer progression by inhibiting the TAZ/PD-L1 signaling pathway [125]. Collectively, these findings suggest that AS’s protective mechanisms involve regulating stem cell properties, delaying cellular senescence, enhancing immunity, and targeting signaling pathways such as PI3K/Akt/FOXO-3a/p27kip1, JAK-2/STAT-3, mTOR, and TAZ/PD-L1. These mechanisms contribute to the protection of various tissues and organs, including the brain, nerves, intestine, and lungs. A detailed overview of the relevant findings from these trials is compiled in Table 1.

## 4. Safety Evaluation of AS

With promising potential for development and broad application prospects, AS’s safety should not be overlooked. Understanding AS’s toxicity and factors related to adverse reaction occurrence can provide valuable insights for its rational clinical use. The dose of AS significantly influences its toxicity and efficacy. Research indicated that a single intravenous dose of AS above one ninth of the maximum non-lethal concentration induced pericardial edema and circulatory defects in a zebrafish larval model. However, AS improved cardiac malformations while restoring cardiac output and hemodynamics at one half of the lowest observed adverse effect level (LOAEL), suggesting significant cardioprotective effects at doses below the LOAEL without adverse events [126].

Furthermore, the duration of administration plays a crucial role in AS toxicity. Short-term administration did not affect bovine oocyte maturation and early embryonic development [127]. However, in rat embryotoxicity studies, the no observed adverse effect level was determined to be 8 mg/kg/day, with 2 mg/kg/day utilized for embryo-fetal development [128]. Additionally, Clark et al. found that AS was embryotoxic when administered at 12 mg/kg/day for more than 12 days but not toxic when given for shorter durations [129]. In a subchronic toxicity study lasting 92 consecutive days, AS at 6 mg/kg caused a decrease in heart rate and suppression of splenic extramedullary hematopoiesis and erythropoiesis in bone marrow, with no observed neurotoxicity [130]. Long-term administration induced lesions in testicular and epididymal tissue in rats but did not affect fertility [131]. These findings suggest that caution should be exercised when using AS at high doses and for prolonged periods due to observed adverse effects under these conditions. Nevertheless, AS’s toxicity in humans remains uncertain, as differences exist between human and animal models, and patient-specific factors such as age, body mass, and height may influence the risk of adverse events [132]. Consequently, close monitoring of patients post-dosing is imperative to minimize the incidence of adverse effects.

## 5. Stability Improvements for AS

AS often requires repeated administration in disease treatment, leading to challenges such as poor patient compliance, heightened disease recurrence, and the emergence of drug resistance. These issues stem primarily from its rapid onset of action and rapid elimination from the body [133]. To address these challenges, Gabriëls et al. developed a sterile liposome suspension encapsulating AS, utilizing lipid components of egg phosphatidylcholine and cholesterol. This suspension, containing AS at a concentration of 1 mg/mL along with 300 mg lipids in a pH 5 buffer, effectively stabilizes AS. In vitro release experiments demonstrated that equilibrium or 30% of the reversibly bound AS was released within 24 h. Importantly, the liposome suspension exhibited stability for at least 10 days at 25 °C [134]. Furthermore, when fortified with iron oxide nanoparticles, the dose of AS could be reduced by 8–10-fold, resulting in reduced parasite recurrence and some extension of anti-malarial efficacy [135]. In summary, sustained-release formulations such as liposomes and nanoparticles offer promising strategies for maintaining steady blood concentrations of AS. This approach not only reduces the frequency of dosing and total administered dose but also holds potential for enhancing the therapeutic efficacy of drugs with short-duration effects that necessitate frequent dosing regimens.

## 6. Conclusions and Future Perspectives

In this review, we comprehensively summarized the physical, chemical, and biological properties of AS, while also evaluating its safety and stability in use. Additionally, we provided a timely overview of AS’s protective effects on organs and tissues both in vivo and in vitro. Collectively, the studies reviewed demonstrate that AS effectively reduces inflammatory cell chemotaxis and inflammatory mediator release by modulating various signaling pathways, including NF-κB, NLRP3, TLR4, PI3K, p42/22 MAPK, PPAR-γ/TGF-β1/Smad, and AMPK/mTORC1/GPX4, across different models of organ injury and disease. Furthermore, AS targets multiple pathways to address fibrosis, including TGF-β1/SMAD2/3, PI3K/Akt, LPS/TLR4/NF-κB, and ROCK1/ATF3. It also corrects metabolic disorders and dysfunction through pathways such as NLRP3/caspase-1/GSDMD and ERK/c-Myc. AS’s protective mechanisms extend to reducing apoptotic protein production and promoting autophagic activity through pathways like PI3K/AKT/mTOR, Wnt/β-catenin, AMPK/SIRT1, ROS-AMPK-mTOR, CaMKKβ-AMPK, AMPK-mTOR-ULK1, p53/p21waf1/cip1h, and PINK1. Moreover, AS enhances stem cell proliferation and differentiation, slows aging, and boosts immunity, further augmenting its protective effects on damaged organs and tissues such as the brain, nerves, heart, lungs, liver, gastrointestinal tract, kidneys, and joints. Taken together, AS demonstrates remarkable protective effects across a spectrum of organs and tissues, suggesting promising prospects for its clinical application.

Oxidative stress has been a hot spot in basic research. The accumulation of a large amount of information has proved that oxidative stress is a key factor indirectly or directly involved in the development of many human diseases. In addition, human organs and tissues are susceptible to the damage caused by oxidative stress. Therefore, the search for effective antioxidants has become a long-term goal in biology, medicine, and other fields. In recent years, AS has received widespread attention due to its significant antioxidant properties. The antioxidant activity of AS is a very crucial step in exerting organ and tissue protection, and it can effectively mitigate excessive oxidative stress, inhibit the expression of multiple oxidative damage markers, enhancing tissue and organ repair. The Nrf2/HO-1 pathway is a key signaling pathway for cellular antioxidative stress. AS can significantly up-regulate the expression of Nrf2 and HO-1, regulate the related antioxidant enzymes to eliminate reactive oxygen species and scavenge free radicals, and exert antioxidative stress, thus alleviating the damage of organs and tissues. In addition, large amounts of ROS and lipid oxidation products can disrupt the operation of the mitochondrial electron transport chain, leading to mitochondrial dysfunction, whereas AS has been found to improve mitochondrial function and dynamics. Continuing to understand the antioxidant mechanism of AS and its modulation of oxidative stress-related genes and signaling pathways may be a useful attempt to broaden the spectrum of antioxidant activity of AS and help us to fight against many human diseases in a more precise way.

Verification of the biological relevance of AS in humans is a crucial step in its clinical application. However, research into the organ and tissue-protective mechanisms of AS remains primarily at the stage of in vitro and animal experiments. The results of experiments in cells and animal models may not be fully representative of the actual effects of AS in humans. There is a clear need for further investment in combining basic research with high-quality clinical translation. Currently, there is insufficient evidence to assess comprehensively the preclinical safety of AS. To ensure safer utilization of AS, it is imperative to employ modern technology to delve deeper into its toxicity mechanisms and determine both safe and toxic doses. In conclusion, future studies should focus on investigating the effects of AS in alleviating organ and tissue damage using larger, more rigorously designed methodological approaches and diverse sample populations. The authors hope that this review will enhance understanding of AS and inspire more scholars to contribute to its study for rescuing organ and tissue injuries. Furthermore, we anticipate that as scientific knowledge advances, the value of AS’s development and application will continue to grow, benefiting mankind across a broader spectrum of fields.

## Figures and Tables

**Table 1:** Organ- and tissue-protective effects of AS.

Organ	Types	Routes	Dosage of Administration	Effects	Molecular Mechanisms	Reference
Brain	In vivo	i.p.	15, 30 mg/kg	↓TNF-α, IL-1β, NF-κB	By inhibiting the NF-κB pathway	Liu et al., 2021 [40]
Brain	In vivo, In vitro	i.p.	20, 50, 70 mg/kg	↑AMPK, mTORC1,GPX4	By inhibiting the AMPK/mTORC1/GPX4 axis	Xie et al., 2023 [50]
Brain	In vivo	i.p.	20, 40, 80, 160 mg/kg	↓TNF-α, IL-6,IL-1β, NF-kB, (TLR)-4, MyD88, MPO	By inhibiting the TLR4/NF-κB pathway	Chen et al., 2021b [43]
Brain	In vivo	-	30 mg/kg	↓TNF-α, IL-6,IL-1β, NF-kB, NLRP3, GFAP, Iba-1 ↑BDNF, GDNF, and NT-3	By inhibiting the NF-κB- NLRP axis	Gugliandolo et al., 2018 [44]
Brain	In vivo, In vitro	i.p.	0.25−2 Μm 0, 5, 15 mg/kg	By inhibiting neuroinflammation and oxidative stress	By regulating antioxidant activity	Huang et al., 2022 [61]
Brain	In vivo, In vitro	i.v.	50 μM	modulated Wnt/β-catenin signaling	By regulating the Wnt/β-catenin pathway	Ismail et al., 2022 [94]
Brain	In vitro	-	37.5 μM	↓IL-1β, MCP-1, IP-10, KC, PI3K/Akt and p44/42 MAPK	By disrupting the ROS-AMPK-mTOR axis	Ding et al., 2023 [106]
Brain	In vivo In vitro	i.p.	0, 0.4, 0.8, 1.6 μmol/L 150 mg/kg	↓PI3K, Akt, FOXO3a, p27kip1	By regulating the PI3K/Akt/FOXO-3a/p27kip1 pathway	Zhang et al., 2020b [119]
Brain	In vitro	-	0.25, 0.5, 1, 2 and 4 µmol/L	↑JAK-2, STAT-3 ↓p-JAK-2, p-STAT-3	By inhibiting the JAK-2/STAT-3 pathway	Luan et al., 2022 [120]
Nerve	In vivo	i.p.	0, 5, 10, or 20 mg/kg	via the ROCK1/ATF3 Axis	By disrupting the ROCK1/ATF3 axis	Wang et al., 2023 [84]
Nerve	In vivo, In vitro	i.p.	0.7 mg/mL 0.15 µg/mL, 0.3 µg/mL	↑p-PI3K, p-AKT, p-mTOR	By regulating the PI3K/AKT/mTOR Axis	Zhang et al., 2023 [88]
Liver	In vivo	i.g	27, 54, 108 mg/kg	↓TNF-α, IL-6,IL-1β, IL-17, NF-kB (p65), (IFN)-γ, ERK, JNK, p38, IkBa, MAPK	By inhibiting the NF-κB and MAPK pathways	Zhao et al., 2017b [45]
Liver	In vitro	-	50 μM	↓TGF-β1, ECM genes ↑matrix-metalloproteinases	By inhibiting the expression of pro-fibrotic genes	Larson et al., 2019 [70]
Liver	In vivo	p.o.	28.8 mg/kg	↓TNF-α,IL-1β, TLR4, ECM, α-SMA, MyD88, TGF-β1, NF-κB p65	By inhibiting the LPS/TLR4/NF-κB pathway	Lai et al., 2015 [74]
Liver	In vivo, In vitro	p.o.	20, 40 mg/kg 40, 80 μM	↓IL-1β, MCP-1, IP-10, KC, PI3K/Akt and p44/42 MAPK	By inhibiting inflammation and collagen fiber deposition	Yuan et al., 2023b [75]
Liver	In vivo, In vitro	i.p.	50 mg/kg	↑LC3, Atg3, Atg5, Atg6/beclin1, Atg12 ↓p62, FTH1, NCOA4	Through ferritin autophagy regulation and induction of HSC iron death	Kong et al., 2019 [83]
Liver	In vivo, In vitro	i.p.	100 mg/kg	↓NDUFB8, UQCRC2	By regulating mitochondrial protein expression	Shen et al., 2021 [85]
Kidney	In vitro	-	15 or 30 μg/mL	↓(TLR)-4, MyD88, NF-kB (p65), NLRP3,ROS, MDA ↑SOD	By inhibiting the TLR4/NF-κB/NLRP3 axis	Sun et al., 2018 [46]
Kidney	In vitro	-	0.01, 0.1, 1 µg/mL	↓TGF-β1, USAG-1, α-SMA ↑BMP-7, E-cadherin	By inhibiting the expression of proteins associated with renal interstitial fibrosis	Zhang et al., 2017b [71]
Kidney	In vivo	i.g,	7.5, 15 mg/kg	↓TNF-α, IL-6,IL-1β, iNOS, NF-kB	By inhibiting the expression of necrosis apoptotic proteins and inflammatory factors	Lei et al., 2021 [95]
Lung	In vivo, In vitro	i.p.	25, 50, 100 mg/kg	↓α-SMA and cyclin D1, TGF-β1, Smad2/3 ↑PPAR-γ,	By regulating the PPAR-γ/TGF-β1/Smad pathway	Pan et al., 2021a [49]
Lung	In vivo	p.o.	10, 30, 100 mg/kg	↓IL-1β, MCP-1, IP-10, KC, PI3K/Akt and p44/42 MAPK ↑NOX2, Nrf2	By inhibiting the PI3K and p42/22 MAPK pathways	Ng et al., 2014 [51]
Lung	In vivo	i.p.	30 mg/kg	↓TNF-α ↑iNOS, NADPH, Nrf2	By activating the Nrf2 pathway	Ho et al., 2012 [56]
Lung	In vivo	i.v.	20, 40 mg/kg	↓MDA, MPO, IL-1β, TNFα, CXCL1, MCP-1, Bax, Cleaved-Caspase3 ↑SOD, Bcl-2, AKT and HO-1	By regulating the levels of genes associated with inflammation, oxidative stress, and apoptosis	Ji et al., 2023 [57]
Lung	In vivo, In vitro	i.v.	10, 20, 40 mg/kg	↓MDA, MPO, IL-1β, TNFα, IL-6 ↑Nrf2 and HO-1	By activating the Nrf2 and HO-1 pathways	Zhao et al., 2017a [58]
Lung	In vivo	i.p.	15 mg/kg	↓COX-2, TNFα, IL-6, NF-κB ↑Nrf2 and HO-1	By activating the Nrf2 and HO-1 pathways	Cao et al., 2015 [59]
Lung	In vitro	-	0, 5, 10, 50 µM	↓GLUT1, enzymes hexokinase and lactate dehydrogenase, c-Myc	By regulating glucose metabolism	Zhang et al., 2022d [62]
Lung	In vivo	i.p.	100 mg/kg	↓TGF-β1, Smad2/3	By reducing the levels of profibrotic molecules	Wang et al., 2015 [72]
Lung	In vivo, In vitro	i.p.	8 µg/mL 100 mg/kg	↓IL-1β, MCP-1, IP-10, KC, PI3K/Akt and p44/42 MAPK	By inhibiting the Notch signaling pathway	Liu et al., 2017 [73]
Lung	In vivo	i.p.	15 mg/kg	↓cl-caspase-3, TNF-α, and IL-6 ↑p-mTOR, p-Akt, and PI3K	By regulating the mTOR/AKT/PI3K pathway	Zhang et al., 2020a [89]
Lung	In vitro	-	100 mM	induced apoptosis through a Bak-mediated intrinsic pathway	By inducing apoptosis through a Bak-mediated intrinsic pathway	Zhou et al., 2012 [90]
Lung	In vivo, In vitro	i.p.	7.5, 15, 25 mg/kg 5, 10, 20 μg/mL	↑SIRT1	By inhibiting apoptosis and neutrophil infiltration	Liu et al., 2023b [91]
Lung	In vivo In vitro	i.p.	40 mg/kg 10 μM	↓TAZ/PD-L1	By inhibiting the TAZ/PD-L1 signaling pathway	Cao et al., 2022 [125]
Ocular	In vivo	s.c.	80 mg/mL	↓TGF- β1/SMAD2/3 and PI3K/Akt, GPX4 ↑Nrf2	By inducing mitochondria-dependent iron death	Liu et al., 2023a [81]
Ocular	In vivo	intravitreal injection	4 μg/μL	By inhibiting CNV and the accompanying fibrotic scar	By inhibiting choroidal neovascularization and modulating mononuclear phagocyte recruitment	Sheibani et al., 2023 [86]
Airway	In vivo	i.p.	30 mg/kg	↓IL-17, IL-12(p40), MCP-1 and G-CSF	By regulating the expression of metabolites	Ho et al., 2014 [63]
Artery	In vivo	i.g,	30 mg/kg	↓HIF-1α, NF-κB	By correcting dysfunction in crucial metabolic pathways	Wang et al., 2022 [64]
Artery	In vivo	i.g	4.5 mg/kg	↓NF-kB, NLRP3, IL-8, IL-1β, caspase-1	By inhibiting the NF-κB/NLRP3 axis	Cen et al., 2023 [47]
Arthrosis	In vitro	-	0.2, 0.4, 0.8 μM	↓ROS ↑p62/Nrf2, NQO1	By activating the p62/Nrf2 pathway	Su et al., 2021 [60]
Knee	In vivo, In vitro	i.g,	10 μM 15, 30, 60 mg/kg	↓IL-1β, MCP-1, IP-10, KC, PI3K/Akt and p44/42 MAPK	By inhibiting mTOR signaling axis	Wan et al., 2019b [104]
Bone	In vivo, In vitro	i.g	1.56, 3.125, 6.25, and 12.5 Μm 10 mg/kg	↓TLR4,TRAF6, PLCγ1-Ca2+-, NFATc1	By inhibiting the TLR4/TRAF6/PLCγ1-Ca axis	Zeng et al., 2019 [52]
Bone	In vitro	-	10 μmol/L	↑p53, p21waf1/cip1	By inducing autophagy	Wan et al., 2019a [108]
Colon	In vivo	i.p.	30 mg/kg	↓TNF-α, IL-6,IL-1β, IκBα, NF-kB (p65) ↑IL-10	By inhibiting the NF-κB pathway	Yin et al., 2020 [48]
Colorectum	In vivo In vitro	i.p.	30 mg/kg 2–15 µM	↓p53, p16, p21, p38MAPK and NF-κB	By suppressing mTOR signaling	Xia et al., 2023 [124]
Intestinal	In vivo In vitro	i.p.	30 mg/kg 2–10 μM	↓mTOR, TNF-α, IL1, and IL6	By downregulating the expression of inflammatory factors	Jia et al., 2023 [123]
Salivary gland	In vivo	i.p.	50 mg/kg	though regulating the PI3K/Akt pathway	By modulating the PI3K/Akt pathway	Zhang et al., 2021 [103]
Retina	In vivo	i.v.	2 or 10 μg	↑Beclin-1, LC3II/I, Beclin-1 expression and LC3II/I ↓p62	Activation of the AMPK/SIRT1 pathway	Li et al., 2021 [105]
The whole body	In vivo, In vitro	i.p.	20 μg/mL 10 mg/kg	↑CaMKKβ, AMPK, ULK1	By modulating the CaMKKβ-AMPK cascade	Liu et al., 2020 [107]
Cervix uterus	In vitro	-	20 μM	↑PINK1	Activation of the PINK1-dependent pathway	Zhang et al., 2018 [109]
Bladder	In vitro	-	0, 25 μM, 50 μM	activating AMPK-mTOR-ULK1 axis	By activating the AMPK-mTOR-ULK1 pathway	Zhou et al., 2020 [110]
Stomach	In vivo	p.o.	50, 150 mg/kg	↓TNF-α, IL-6,IL-1β, NF-kB (p65),TBARS and MPO ↑GSH, SOD,	By inhibiting the NF-κB pathway	Verma & Kumar, 2016 [41]
Pancreas	In vitro	-	0.2–3.0 uM	via inhibition of the NLRP3/caspase-1/GSDMD	By inhibiting the NLRP3/caspase-1/GSDMD pathway	Yuan et al., 2022 [65]

## Data Availability

No data was used for the research described in the article.
